# A Novel Range Compression Algorithm for Resolution Enhancement in GNSS-SARs

**DOI:** 10.3390/s17071496

**Published:** 2017-06-25

**Authors:** Yu Zheng, Yang Yang, Wu Chen

**Affiliations:** Department of Land Surveying and Geo-informatics, The Hong Kong Polytechnic University, Hung Hom, Kowloon, Hong Kong, China; yyang1018@gmail.com (Y.Y.); wu.chen@polyu.edu.hk (W.C.)

**Keywords:** GNSS-SAR, global navigation satellite system, synthetic aperture radar, range compression, range resolution

## Abstract

In this paper, a novel range compression algorithm for enhancing range resolutions of a passive Global Navigation Satellite System-based Synthetic Aperture Radar (GNSS-SAR) is proposed. In the proposed algorithm, within each azimuth bin, firstly range compression is carried out by correlating a reflected GNSS intermediate frequency (IF) signal with a synchronized direct GNSS base-band signal in the range domain. Thereafter, spectrum equalization is applied to the compressed results for suppressing side lobes to obtain a final range-compressed signal. Both theoretical analysis and simulation results have demonstrated that significant range resolution improvement in GNSS-SAR images can be achieved by the proposed range compression algorithm, compared to the conventional range compression algorithm.

## 1. Introduction

The passive Global Navigation Satellite System (GNSS)-based Synthetic Aperture Radar (SAR), also known as GNSS-SAR, is a technique for remote sensing that has been developing in recent years [1,2]. Unlike conventional SAR techniques, the GNSS-SAR is a passive SAR receiver which uses the signals from Global Navigation Satellite Systems (GNSSs) such as the global positioning system (GPS), Galileo, GLONASS or Beidou for transmission of opportunity. Due to the fact that there is no need to construct a SAR transmitter, the GNSS-SAR has a higher flexibility and implies lower expenses than a conventional SAR under various applications. However, low range resolution is one of the main problems that affects the current development of GNSS-SARs [1,2,3,4,5].

With a conventional GNSS-SAR imaging algorithm that includes both range and azimuth compression, range resolution is determined by GNSS signal bandwidth and bi-static angle for sensing, while azimuth resolution is determined by Doppler frequency shift [1,2,4,6,7,8,9,10,11,12,13,14,15,16,17]. However, when the system is considered as a quasi-monostatic case, in which the bi-static angle is $0^{\circ }$ and range and azimuth domain are orthogonal, range resolution is mainly decided by the GNSS signal bandwidth [1,2,4,7,8,9,10,11,12,13,14,15,16,17]. If shape factors of a wave form are not considered, potential range resolution identically equals the reciprocal of doubled signal bandwidth value. For GNSS signals, the bandwidth value is equal to the pseudo-random noise (PRN) code chip rate [1,2]. For instance, because the chip rate of GPS Coarse Acquisition Code (C/A) code signal is 1.023 MHz, the potential range resolution is obtained at the level of 150 m [1,2,16]. A GLONASS P code signal, where the chip rate is 5.11 MHz, with GPS P code signal and Beidou signal, where the chip rate is 10.23 MHz, were used in works [1,2,4,6,7,8,9,10,12,13,17], respectively. The potential range resolution for [1,2,10,12,13] was obtained at the level of 30 m and 15 m in [4,6,7,8,9,17]. Galileo signals were employed in [11] (single Galileo E5) and [14,15] (joint Galileo E5), in which a single Galileo E5 signal could provide potential range resolution at the level of 15 m with a chip rate of 10.23 MHz and 3 m for joint Galileo E5 signal with a joint chip rate of 51 MHz based on a bandwidth extension technique [18]. In total, according to [1,2,6,7,8,9,10,11,12,13,14,15,16], as we can see, use of GNSS signals with relatively higher chip rates is currently preferred, because relatively improved range resolutions can be obtained. Looking at the azimuth resolution of the GNSS-SAR, the level less than 1 m can be potentially achieved for moving receiver [10], while for fixed receiver, a level 3–4 m can be potentially obtained by 300 s integration [11]. Meanwhile, besides the aforementioned works, spatial resolution enhancement of the GNSS-SAR was studied in [3,4,5]. Typically, in works [4,5], the spatial resolution improvements were carried out based on multi-static image processing method with clean techniques [19,20] for extracting a scene-scattering center, in which both range and azimuth resolutions were improved. Based on GLONASS encrypted precision code (P code) signal and Beidou signal as source of opportunity as examples, spatial resolutions in [4,5] were improved to a level of 6–7 m.

It can be seen that under a conventional GNSS-SAR range compression algorithm, with a given bi-static topology, the signal chip rate is the main factor that restricts improvements of range resolution. Although a multi-statistic image processing method with clean techniques [4,5] can enhance spatial resolution of various GNSS-SARs, a shortcoming of the approaches is that the methods are time consuming as they are applied after generating multiple full preliminary GNSS-SAR images.

In contrast to [4,5,13], the objective in this research is to enhance range resolution in the GNSS-SAR imaging procedure. To achieve the objective, the main contribution in this paper is to propose a new range compression algorithm for GNSS-SAR imaging signal processing to improve range-compressed resolution. In the proposed algorithm, within each azimuth bin, at first, the received intermediate frequency (IF)-reflected GNSS signal is correlated with the synchronized direct baseband GNSS signal at the range domain for each azimuth bin for performing range compression. Then spectrum equalization [14] is applied to suppress side lobes of the compressed result for achieving the final range-compressed signal. The theoretical derivation and simulation results show that the proposed range compression algorithm can improve range resolution in the GNSS-SAR significantly, compared to conventional range compression algorithm. Also, the proposed range compression algorithm is less time consuming than multi-static image processing [4,5] for enhancing range resolution as there is no necessity for obtaining multiple full preliminary GNSS-SAR images.

The rest of the paper is organized as follows. Resolution of the conventional range compression algorithm is analyzed in Section 2. Resolution of the proposed range compression algorithm is analyzed in Section 3. The simulation tests are provided in Section 4. Section 5 discusses the associated issues of this research as well as future developments. Section 6 provides the final concluding remarks of the paper.

## 2. Resolution of the Conventional Range Compression Algorithm

**Figure 1:**
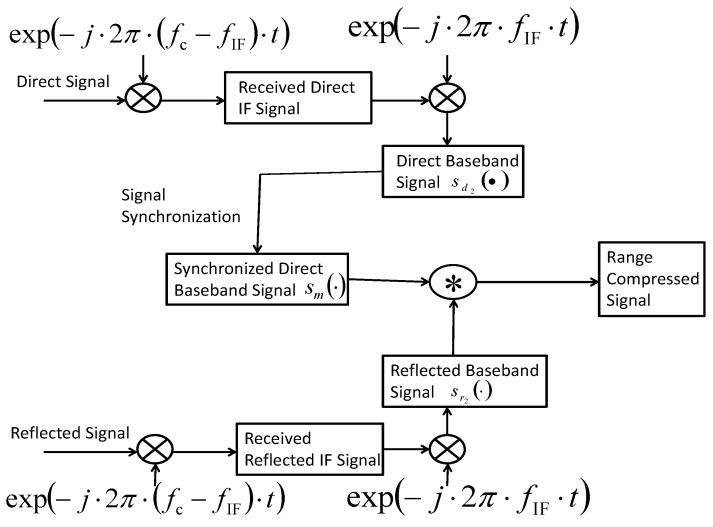
The flow diagram of the conventional range compression algorithm.

Based on the analysis in [1,2,4,5,6,7,12,13,14,15], the flow diagram of the conventional range compression algorithm at the GNSS-SAR receiver can be illustrated as Figure 1.

In Figure 1, under the conventional range compression algorithm, both direct and reflected signals are quadrature, converted to an intermediate frequency (IF) band by multiplying the component $\exp\left(-j2\pi \cdot \left(f_{c}-f_{\mathrm{IF}}\right)\cdot t\right)$ at first, where $f_{c}$ denotes the transmission frequency, $f_{\mathrm{IF}}$ denotes the intermediate frequency (IF) of the employed GNSS receiver, ⊗ denotes arithmetic multiplication and ⊛ denotes arithmetic correlation.

The received IF signals (both direct and reflected signal) are further down-converted to baseband by multiplying the component $\exp\left(-j2\pi \cdot f_{\mathrm{IF}}\cdot t\right)$. The down-converted direct baseband signal can then be expressed as:

(1) $$\begin{array}{cc} s_{d_{2}}\left(t,u\right) & =A_{d}\left(t,u\right)C\left[t-\tau \left(u\right)\right]D\left[t-\tau \left(u\right)\right]\\ & \;\times \exp\left(j\left(2\pi f_{d}\left(u\right)\cdot t+\phi_{d}\left(u\right)\right)\right)\\ & \;+n_{d}\left(t,u\right) \end{array}$$

and the reflected baseband signal can be expressed as:

(2) $$\begin{array}{cc} s_{r_{2}}\left(t,u\right) & =A_{r}\left(t,u\right)C\left[t-\tau \left(u\right)-\tau_{R}\left(u\right)\right]\\ & \;\times D\left[t-\tau \left(u\right)-\tau_{R}\left(u\right)\right]\\ & \;\times \exp\left(j\left(2\pi f_{d}\left(u\right)\cdot t+\phi_{r}\left(u\right)\right)\right)\\ & \;+n_{r}\left(t,u\right). \end{array}$$

where $A_{d}$ and $A_{r}$ denote magnitudes of the direct and reflected signals, respectively; $C\left(\cdot \right)$ denotes PRN code; $D\left(\cdot \right)$ denotes the navigation bits; *t* denotes fast time [1,2], which represents the range domain and is constrained by one GNSS PRN code period; *u* denotes slow time [1,2], which represents the azimuth domain and is limited by the duration for performing aperture synthesizing; τ denotes the received direct signal code delay relative to the transmitted signal; $\tau_{R}$ denotes the received reflected signal code delay relative to the direct signal; $f_{d}$ denotes Doppler frequency; $\phi_{d}$ denotes direct signal phase, and $\phi_{r}$ denotes reflected signal phase, which can be regarded as constant values within each range bin; *j* denotes the imaginary unit; $n_{d}$ denotes the background noise at direct channel and $n_{r}$ denotes the background noise at a reflected channel.

Thereafter signal synchronization [1,2,12] based on down-converted baseband direct signal as (1) is performed. A noise-free replica $s_{m}$ of baseband direct signal is generated using the parameter direct code delay τ, Doppler frequency $f_{d}$, and direct signal phase $\phi_{d}$ tracked from the synchronization procedure and served as an imaging matched filter. The replica can be mathematically modeled as follows:

(3) $$s_{m}\left(t,u\right)=C\left[t-\tau \left(u\right)\right]D\left[t-\tau \left(u\right)\right]\times \exp\left(j\left(2\pi f_{d}\left(u\right)\cdot t+\phi_{d}\left(u\right)\right)\right).$$

Range compression is conducted through correlating baseband reflected signal $s_{r_{2}}$ with imaging matched filter $s_{m}$ at each range domain, with a duration constrained by the PRN code period although it is not necessarily equal. The range-compressed result with respect to the noise absence term can be expressed as follows:

(4) $$\begin{array}{c} s_{r_{2}}⊛s_{m}^{*}\\ \;=A_{r}\cdot \Lambda \left(t-\tau \left(u\right)-\tau_{R}\left(u\right)\right)\times \exp\left(j\left(\phi_{r}\left(u\right)-\phi_{d}\left(u\right)\right)\right) \end{array}$$

where $\Lambda \left(\cdot \right)$ indicates triangle function and its duration is determined by the PRN code chip rate of the GNSS signal; and * denotes the conjugate. In (4), because $s_{r_{2}}$ and $s_{m}$ are with the same frequency $f_{d}$, the frequency component after performing range correlation for the compression is canceled. Assuming the chip rate of PRN code $C\left(\cdot \right)$ is *B*, then the effective pulse duration of the triangle function $\Lambda \left(\cdot \right)$ is derived as $0.586\cdot \frac{1}{B}$, where 0.586 represents shape factor of waveform [2]. Because the terms $A_{r}$ and $\exp\left(j\left(\phi_{r}\left(u\right)-\phi_{d}\left(u\right)\right)\right)$ are constants with respect to *t*, the duration of (4) is determined by the term $\Lambda \left(\cdot \right)$. Thus, the attainable range resolution with respect to pulse $\Lambda \left(\cdot \right)$ duration can be expressed as [1,2,4,6,7,9,10,11,12,13,14,17]

(5) $$\delta_{R_{1}}=0.586\cdot \frac{c}{\cos\left(\beta /2\right)\cdot B}$$

where *c* denotes the speed of light, β represents bi-static angle and $\delta_{R_{1}}$ represents the achievable range resolution by the conventional algorithm. According to (5), it can be seen that for the conventional range compression algorithm in the GNSS-SAR, if bi-static topology is given prior, the range resolution is limited by signal chip rate, and improvement can only be accomplished by employing the GNSS signals with a relatively higher PRN code chip rate as expected by end users.

## 3. Resolution of the Proposed Range Compression Algorithm

**Figure 2:**
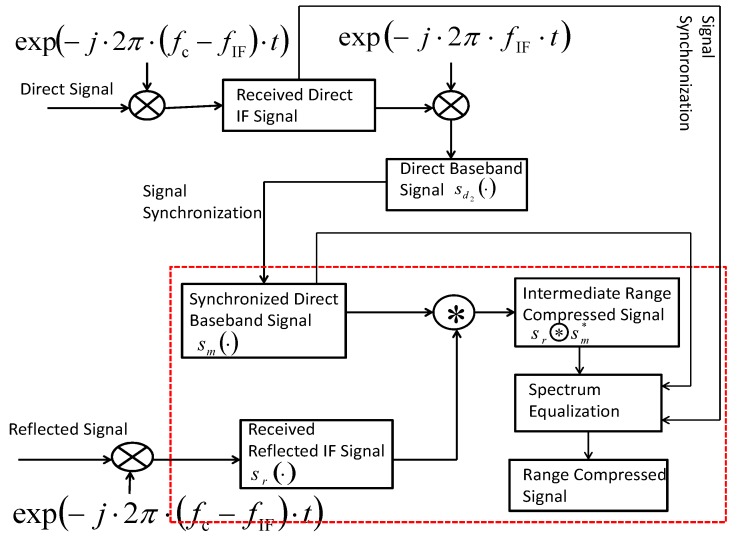
The flow diagram of the proposed range compression algorithm.

According to [21], we can derive that for the digital communication signals structured similarly or the same as GNSS signals, if the two signals for performing correlation differ in frequency, pulse duration of the correlated result will be sharpened in the main lobe, compared to the case with the same frequencies. Inspired by this, to develop a GNSS-SAR imaging algorithm for improving range resolution, a new range compression algorithm is proposed, which is modeled in Figure 2.

In Figure 2, the signals (both direct and reflected) are converted to the IF band at the front-end GNSS receiver as well. However, in contrast to conventional range compression algorithms, at the first step, the proposed new algorithm directly uses the received reflected GNSS IF signal to correlate with the synchronized direct base-band signal $s_{m}$ in the range domain for performing range compression. The reflected GNSS IF signal $s_{r}\left(\cdot \right)$ is expressed as follows:

(6) $$\begin{array}{cc} s_{r}\left(t,u\right) & =A_{r}\left(t,u\right)C\left[t-\tau \left(u\right)-\tau_{R}\left(u\right)\right]\\ & \;\times D\left[t-\tau \left(u\right)-\tau_{R}\left(u\right)\right]\\ & \;\times \exp\left(j\left(2\pi \left(f_{\mathrm{IF}}+f_{d}\left(u\right)\right)\cdot t+\phi_{r}\left(u\right)\right)\right)\\ & \;+n_{r}\left(t,u\right) \end{array}$$

and the intermediate range-compressed result (i.e., the result after performing $s_{r}⊛s_{m}^{*}$) can be expressed as follows:

(7) $$\begin{array}{c} s_{r}⊛s_{m}^{*}\\ \;=A_{r}\cdot \Lambda \left(t-\tau \left(u\right)-\tau_{R}\left(u\right)\right)\\ \;\;\times \exp\left(j\left(2\pi f_{\mathrm{IF}}\cdot t+\left(\phi_{r}\left(u\right)-\phi_{d}\left(u\right)\right)\right)\right). \end{array}$$

**Figure 3:**
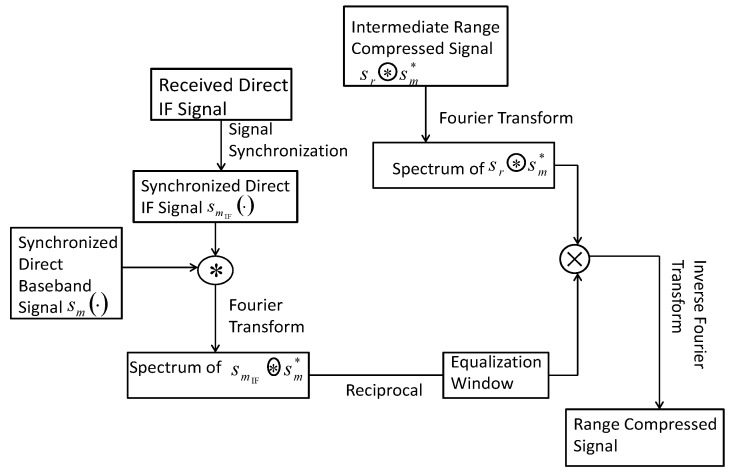
The flow diagram of ‘spectrum equalization’ module of the proposed range compression algorithm.

Based on the intermediate range-compressed result (7), to suppress the compressed side lobes, spectrum equalization [14] is performed. When applying spectrum equalization technique in this paper, the detailed procedure in the module ‘spectrum equalization’ in Figure 2 can be further presented as Figure 3.

As we can see in Figure 3, Fourier transform of intermediate range-compressed signal as (7) is conducted. The transformed result is expressed as follows:

(8) $$\begin{array}{c} F\left[s_{r}⊛s_{m}^{*}\right]\\ \;=\int_{-\frac{T_{s}}{2}}^{\frac{T_{s}}{2}}A_{r}\cdot \Lambda \left(t-\tau \left(u\right)-\tau_{R}\left(u\right)\right)\\ \;\;\times \exp\left(j\left(2\pi f_{\mathrm{IF}}\cdot t+\left(\phi_{r}\left(u\right)-\phi_{d}\left(u\right)\right)\right)\right)\\ \;\;\times \exp\left(-j\omega \cdot t\right)dt\\ \;=A_{r}\cdot \exp\left(j\left(\phi_{r}\left(u\right)-\phi_{d}\left(u\right)\right)\right)\\ \;\;\times {\mathrm{sinc}}^{2}\left(2\pi \cdot f_{\mathrm{IF}}-\omega \right) \end{array}$$

where $T_{s}$ denotes the considered duration for performing range compression and ω denotes the frequency range of the triangle function $\Lambda \left(\cdot \right)$ in (7) with an interval of $\left[-2\pi \cdot B,2\pi \cdot B\right]$. Meanwhile, the spectrum equalization window is designed, which is based on the reciprocal of the spectrum with respect to the correlation between the synchronized direct IF signal $s_{m_{\mathrm{IF}}}$ and the synchronized direct base-band signal $s_{m}$. In Figure 3, the synchronized direct IF signal is given as follows:

(9) $$\begin{array}{cc} s_{m_{\mathrm{IF}}}\left(t,u\right) & =C\left[t-\tau \left(u\right)\right]D\left[t-\tau \left(u\right)\right]\\ & \;\times \exp\left(j\left(2\pi \left(f_{\mathrm{IF}}+f_{d}\left(u\right)\right)\cdot t+\phi_{d}\left(u\right)\right)\right) \end{array}$$

the correlated result between $s_{m_{\mathrm{IF}}}$ and $s_{m}$ is given as:

(10) $$\begin{array}{c} s_{m_{\mathrm{IF}}}⊛s_{m}^{*}\\ \;=\Lambda \left(t-\tau \left(u\right)\right)\times \exp\left(j2\pi f_{\mathrm{IF}}\cdot t\right) \end{array}$$

and the spectrum of the correlated result is identical to the Fourier transform of (10), which can be expressed as:

(11) $$F\left[s_{m_{\mathrm{IF}}}⊛s_{m}^{*}\right]={\mathrm{sinc}}^{2}\left(2\pi \cdot f_{\mathrm{IF}}-\omega \right).$$

Then the equalization window is designed as follows:

(12) $$W=\left\{\begin{array}{cc} \frac{1}{F\left[s_{m_{\mathrm{IF}}}⊛s_{m}^{*}\right]}=\frac{1}{{\mathrm{sinc}}^{2}\left(2\pi \cdot f_{\mathrm{IF}}-\omega \right)}, & \mathrm{when}\mathrm{frequency}\epsilon \left[f_{\mathrm{IF}}-B,f_{\mathrm{IF}}+B\right]\\ 0, & \mathrm{Otherwise} \end{array}\right..$$

The key step of spectrum equalization is performed as follows:

(13) $$\begin{array}{c} F\left[s_{r}⊛s_{m}^{*}\right]\times W\\ =\left\{\begin{array}{cc} A_{r}\cdot \exp\left(j\left(\phi_{r}\left(u\right)-\phi_{d}\left(u\right)\right)\right), & \mathrm{when}\mathrm{frequency}\epsilon \left[f_{\mathrm{IF}}-B,f_{\mathrm{IF}}+B\right]\\ 0, & \mathrm{otherwise} \end{array}\right.. \end{array}$$

The equalized result is a rectangular function at frequency domain, where the rising edge appears at the frequency $f_{\mathrm{IF}}-B$ and the falling edge appears at the frequency $f_{\mathrm{IF}}+B$. Due to the fact that spectrum equalization is conducted at frequency domain, side lobes of the reflected signals at different range positions can be suppressed simultaneously.

To obtain the final range-compressed signal, inverse Fourier transform based on the spectrum equalized result shown in (13) is conducted. To extract the sharper pulse duration component, the lower frequency component $f_{\mathrm{IF}}-B$ is filtered out. The final range-compressed signal module of Figure 2 and Figure 3 with regard to noise absence term is expressed as follows:

(14) $$\begin{array}{c} F^{-1}\left\{F\left[s_{r}⊛s_{m}^{*}\right]\times W\right\}\\ \;=A_{r}\cdot \exp\left(j\left(\phi_{r}\left(u\right)-\phi_{d}\left(u\right)\right)\right)\cdot \left(f_{\mathrm{IF}}+B\right)\\ \;\;\times \mathrm{sinc}\left[2\pi \cdot \left(f_{\mathrm{IF}}+B\right)\cdot \left(t-\tau \left(u\right)-\tau_{R}\left(u\right)\right)\right]. \end{array}$$

In (14), the pulse duration is determined by the component $f_{\mathrm{IF}}+B$ of the $\mathrm{sinc}\left(\cdot \right)$ function term, and can be derived as $\frac{1}{f_{\mathrm{IF}}+B}$. Thus, the attainable range resolution with regard to pulse duration is expressed as:

(15) $$\delta_{R_{2}}=0.586\cdot \frac{c}{\cos\left(\beta /2\right)\cdot \left(f_{\mathrm{IF}}+B\right)}$$

where $\delta_{R_{2}}$ denotes the range resolution obtained by the proposed algorithm. It can be seen that (15) is $\frac{1}{1+f_{\mathrm{IF}}/B}$ times superior than the range resolution (5) provided by the conventional range compression algorithm. Meanwhile, from (14), we can see that the reflected phase information $\phi_{r}-\phi_{d}$ is still preserved.

For selecting the IF value in the proposed algorithm, sampling frequency of the employed GNSS receiver should be taken into consideration. Denoting the sampling frequency of the GNSS receiver as $f_{s}$, according to sampling theory [22], the condition $f_{\mathrm{IF}}+B\leq \frac{1}{2}f_{s}$ should be satisfied. To make the proposed algorithm effective, the condition $f_{\mathrm{IF}}+B>B$ should be satisfied at the same time as well. Therefore, the determination of $f_{\mathrm{IF}}$ value should satisfy the following constraint:

(16) $$0<f_{\mathrm{IF}}\leq \frac{1}{2}f_{s}-B.$$

Finally, azimuth compression is conducted for forming the full GNSS-SAR image based on (14) with different differential phase value $\phi_{r}\left(u\right)-\phi_{d}\left(u\right)$ in the azimuth domain [2,12].

## 4. The Simulation Experiment

To test the proposed algorithm for enhancing range resolution, simulations of the GNSS-SAR based on the standard GPS C/A code signal receiver configuration are carried out in this section as an example. We consider that the system works in quasi-monostatic mode where range and azimuth directions are orthogonal. Thus, the bi-static angle β can be considered as zero [2]. Based on the system mode, range resolutions of the conventional algorithm and the proposed algorithm are expressed as follows, respectively,

(17) $$\delta_{R_{1}}^{{}^{\prime }}=0.586\cdot \frac{c}{B}$$

and

(18) $$\delta_{R_{2}}^{{}^{\prime }}=0.586\cdot \frac{c}{\left(f_{\mathrm{IF}}+B\right)}.$$

The parameter values of the standard GPS C/A code receiver are given in Table 1.

**Figure 4:**
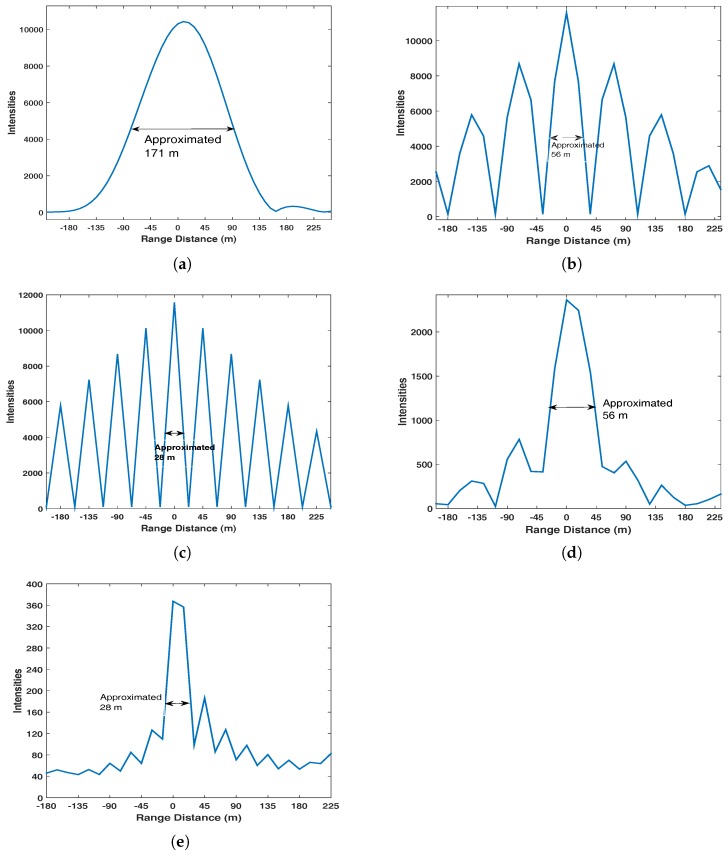
(**a**) Range-compressed pulse based on the conventional range compression algorithm; (**b**) Intermediate range-compressed result based on the proposed range compression algorithm with $f_{{\mathrm{IF}}_{1}}=2.092\;\mathrm{MHz}$; (**c**) Intermediate range-compressed pulse based on the proposed range compression algorithm with $f_{{\mathrm{IF}}_{2}}=5.115\;\mathrm{MHz}$; (**d**) Final range-compressed result based on the proposed range compression algorithm with $f_{{\mathrm{IF}}_{1}}=2.092\;\mathrm{MHz}$; (**e**) Final range-compressed result based on the proposed range compression algorithm with $f_{{\mathrm{IF}}_{2}}=5.115\;\mathrm{MHz}$.

Constrained by the sampling frequency value in Table 1, two different IF frequencies $f_{{\mathrm{IF}}_{1}}=2.092$ MHz and $f_{{\mathrm{IF}}_{2}}=5.115$ MHz are employed in the simulation tests. Theoretically based on (17), range resolution for the conventional algorithm can be achieved at the level of 171 m. For the proposed algorithm, based on (18), range resolution can be obtained at the levels of 56 m and 28 m with $f_{{\mathrm{IF}}_{1}}$ and $f_{{\mathrm{IF}}_{2}}$, respectively. Firstly the verdict will be verified by the result with respect to the range-compressed pulse [3,14] of the conventional range compression algorithm, intermediate range-compressed result and the final range-compressed result (the result after performing spectrum equalization) of the proposed range compression algorithm, which are shown in Figure 4.

From Figure 4, we can see that based on a standard GPS C/A code signal receiver, the main lobe of the range-compressed result based on the proposed algorithm is around 3 and 6 times thinner than the conventional algorithm with $f_{{\mathrm{IF}}_{1}}$ and $f_{{\mathrm{IF}}_{2}}$, respectively. In the proposed algorithm, it can be seen that the side-lobes can be significantly suppressed by performing spectrum equalization.

**Figure 5:**
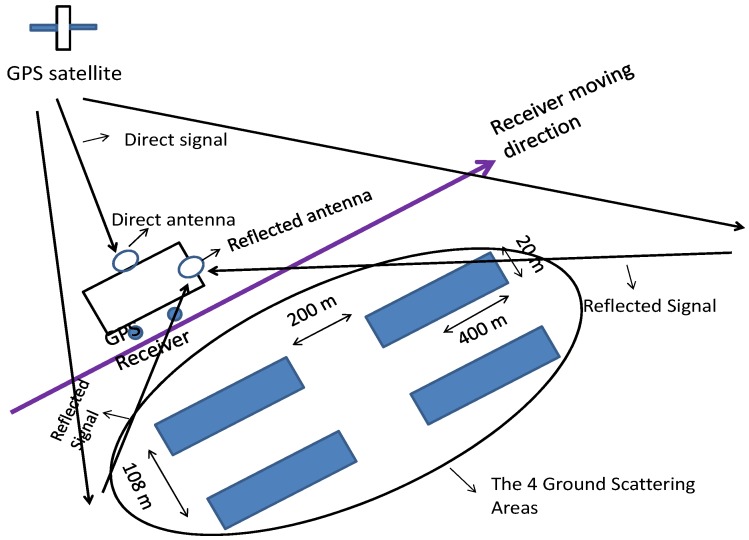
The simulation scenario.

Thereafter, to further verify the proposed range compression algorithm, a simulation test is carried out. The simulation experiment is set out as shown in Figure 5. In the experiment, a moving receiver case and a short-range geometry is considered, where the quasi-monostatic assumption is held.

**Figure 6:**
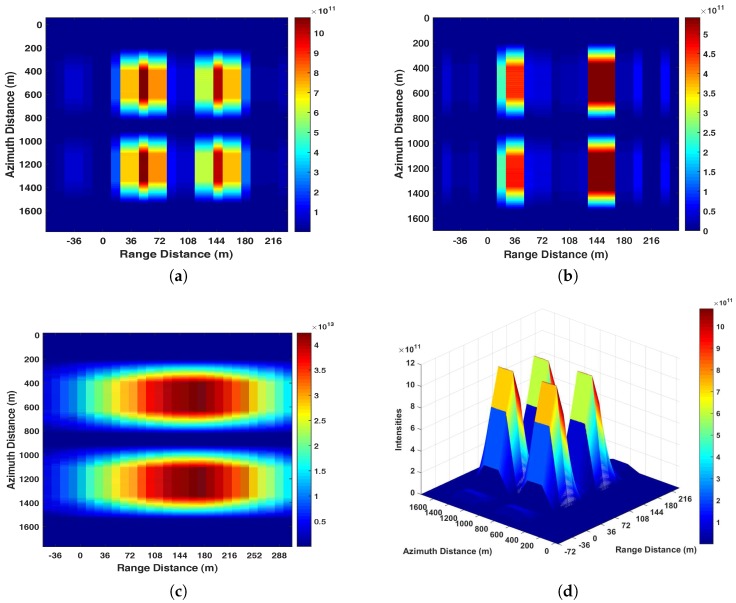

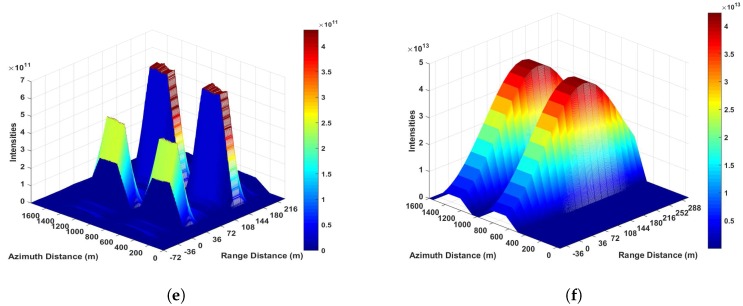
(**a**) GNSS-SAR image generated by the proposed range compression algorithm with $f_{{\mathrm{IF}}_{1}}=2.092\;\mathrm{MHz}$; (**b**) GNSS-SAR image generated by the proposed range compression algorithm with $f_{{\mathrm{IF}}_{2}}=5.115\;\mathrm{MHz}$; (**c**) GNSS-SAR image generated by the conventional range compression algorithm; (**d**) Three-dimensional image of (**a**); (**e**) Three-dimensional image of (**b**); (**f**) Three-dimensional image of (**c**).

In Figure 5, four strong reflection surfaces with length of 400 m and width of 20 m are arranged with 200 m along the azimuth direction and 108 m with the range direction. The direct and reflect signal antennae are moving along the azimuth direction with a constant speed to perform synthetic aperture. The GPS data are simulated using parameters listed in Table 1. Based on the considered scenarios, the GNSS-SAR images (both 2-D and 3-D view) generated by both the proposed range compression algorithm and the conventional range compression algorithm are shown in Figure 6.

As can be seen in Figure 6a,b (Figure 6d,e), due to the fact that the proposed range compression algorithm can offer a superior range resolution, the four scattering areas in Figure 5 can be well separated. Through the comparisons, Figure 6b (Figure 6e) has a less range ambiguity because a higher IF value is employed at the GPS receiver. In Figure 6c (Figure 6f), the two scatters located at different range positions cannot be separated on the GNSS-SAR image with the conventional range compression algorithms, as the resolution of this algorithm is around 171 m according to (17) with B=1.023 MHz.

In summary, the simulation results in this section have demonstrated that the proposed range compression algorithm can provide a superior range resolution to the conventional range compression algorithm.

Furthermore through tests, the proposed range compression algorithm is also applicable for the GNSS-SAR receiver based on the other GNSS signals of opportunity. In the GNSS receiver, the IF value is typically higher than baseband frequency (which equals the PRN code chip rate) of the corresponding compatible signal. For instance, in the considered GPS C/A code signal receiver in this simulation, $f_{{\mathrm{IF}}_{1}}$ and $f_{{\mathrm{IF}}_{2}}$ values are obviously higher than baseband frequency of GPS C/A signal. Therefore, a superior range resolution should be achieved by employing the proposed range compression algorithm. However, because the GNSS receivers differ in the PRN code types and IF values for signal reception, the achievable range resolutions after improving will be different as well.

## 5. Discussion

### 5.1. Discussion on the Associated Issues

Based on the theoretical derivation and simulation experiment, it has been demonstrated that the proposed range compression algorithm enhances range resolution significantly more than the conventional range compression algorithm during the GNSS-SAR imaging procedure. For instance, in joint Galileo E5 signal receiver-based GNSS-SARs (where the original range resolution is 3 m [14,15]), the range resolution can be potentially obtained at a level less than 1 m using the proposed range compression algorithm. For the bistatic GNSS-SAR where range and azimuth direction are not orthogonal [3,6], although imaging performance will be largely impacted by other factors, such as the angle between range and azimuth direction, besides range and azimuth resolution indicators, using the proposed range compression algorithm for obtaining superior range resolution value can still help in achieving higher quality images. However, according to Figure 4, it can be seen that the scene illumination level decreases with respect to $f_{\mathrm{IF}}$ values. This is because when performing spectrum equalization, signal-to-noise ratio (SNR) will be decreased with respect to the selected cutoff frequency [14], which is the main associated problem of the proposed range compression algorithm compared to multi-static image processing [4,5]. Since the mathematical expression of SNR loss caused by spectrum equalization is introduced in [14], it is omitted at here. According to the corresponding mathematical expression in [14], combining the experimental results in this paper, it can be seen that spectrum equalization causes around 5 times more SNR degradation and 30 times more SNR degradation with $f_{{\mathrm{IF}}_{1}}$ and $f_{{\mathrm{IF}}_{2}}$ before performing azimuth compression, respectively. Since GNSSs are low-equivalent isotropically radiated power (EIRP) sources, losses in SNR cannot be so easily tolerated. However, using lengthened integration when performing imaging will help to improve scene illumination level. In some specific operative scenarios such as the permanent monitoring of bridges or mines by employing a close receiver, the losses in SNR can be acceptable.

Concerning another associated issue, time consumption, we study it based on judging the load with regard to the number of operations. For multi-static image processing [4,5] for improving resolution, we can easily see that the method is more time consuming than the proposed algorithm because it is applied based on generating multiple full preliminary GNSS-SAR images. Also, the availability of a multiple perspective exploiting multiple transmitter is not intended for the range resolution improvement only, but provides the capability of exploiting spatial diversity in different ways. Therefore we do not compare the works [4,5] with this paper in detail. Comparing the proposed range compression algorithm with a conventional range compression algorithm, the former has a higher computational load due to the fact that spectrum equalization is used. To quantify the computational load, the corresponding analysis is provided as follows. Assume for received GNSS signal (both direct and reflected), there are *N* samples at the range domain, *M* samples at the azimuth domain (each azimuth sample represents 1 millisecond) and azimuth resolution cell size is $M_{s}$ samples. For GNSS-SAR imaging based on the conventional range compression algorithm, there is $O\left(N^{2}\times M\right)$ number of operations throughout the range compression state, $O\left(M_{s}\times M\times N\right)$ number of operations for azimuth compression per resolution cell and $O\left(\left(M-M_{s}\right)\times M_{s}\times M\times N\right)$ number of operations throughput whole azimuth compression state. Thus, the accumulated number of operations during imaging is:

(19) $$O\left(N^{2}\times M+\left(M-M_{s}\right)\times M_{s}\times M\times N\right).$$

For GNSS-SAR imaging based on the proposed range compression algorithm, for range processing of each azimuth bin, there additionally exists $O\left(N^{2}\right)$ number of operations for performing (10), $O\left(N\cdot \log\left(N\right)\right)$ number of operations for performing (8), (11) and (14) respectively, as well as $O\left(N\right)$ number of operations for performing (13). Fourier transform does not change the sample quantity. The numbers of operations for designing a spectrum equalization window is very small, which can be neglected when compared with imaging computations. For azimuth processing, the numbers of operations are the same as GNSS-SAR imaging based on the conventional range compression algorithm. Therefore the accumulated number of operations during imaging can be derived as:

(20) $$O\left(\left(2N^{2}+3N\cdot \log\left(N\right)+N\right)\times M+\left(M-M_{s}\right)\times M_{s}\times M\times N\right)$$

where $O\left(\left(2N^{2}+3N\cdot \log\left(N\right)+N\right)\times M\right)$ is for range processing. It can be seen that the value of (20) is higher than (19). This indicates that the proposed range compression algorithm has a higher processing delay than the conventional range compression algorithm.

Although the proposed range compression algorithm has been demonstrated in GNSS-SAR in this paper, due to the signal structure difference, the feasibility of the proposed algorithm in the passive radar system using other signals of opportunity besides GNSS still needs to be further studied.

### 5.2. Future Development

Based on the analysis above, in our future work, firstly, we will test the feasibility of the proposed range compression algorithm in the passive radar system using other sources of opportunity besides GNSS, and modify the proposed algorithm based on the corresponding signal structures. Secondly, we would like to develop a mechanism for GNSS-SAR receivers for trade-off among range resolution, range-compressed SNR degradation and compressed delay together with the corresponding field experimental studies.

## 6. Conclusions

In this paper, a novel range compression algorithm for enhancing the resolution of the GNSS-SAR is proposed. In range compression, the received reflected IF GNSS signal is correlated with the synchronized direct baseband signal in the range domain for each azimuth bin. Then, side lobes of the range-compressed result are suppressed by a proper designed spectrum equalization window. Both theoretical derivation and simulation results have demonstrated that the proposed range compression algorithm can provide a superior range resolution compared to the conventional range compression algorithm in the GNSS-SAR. Moreover, the proposed algorithm is less time-consuming than multi-static image processing [4,5] due to the fact that it is applied without the necessity for generating multiple full preliminary images, and is in general applicable in various GNSS-SARs. At the same time, a non-negligible main limitation of the proposed range compression algorithm is the loss in SNR. However, lengthened integration during imaging will help to enhance scene illumination level, and under the specific operative scenarios such as the permanent monitoring of bridges or mines using a close GNSS-SAR receiver, the SNR losses can be acceptable.

## Figures and Tables

**Table 1:** The parameter values of the standard global positioning system (GPS) receiver configuration-based Global Navigation Satellite System-Synthetic Aperture Radar (GNSS-SAR). PRN: pseudo-random noise; C/A: Coarse Acquisition Code.

Parameters	Values
Supported signals type	GPS C/A code signal
PRN code chip rate *B*	1.023 MHz
Signal transmission frequency $f_{c}$	1575.42 MHz (L1 band)
Signal transmission speed *c*	$3\times 10^{8}$ m/s
The duration of	
each code period	1 ms
Sampling frequency	16.368 MHz

## Abbreviations

The following abbreviations are used in this manuscript:

GNSS	Global Navigation Satellite System
GPS	global positioning system
SAR	Synthetic Aperture Radar
IF	intermediate frequency
SNR	signal to noise ratio
C/A code	Coarse Acquisition Code
P code	Encrypted Precision Code
EIRP	equivalent isotropically radiated power
PRN	pseudo-random noise
